# Measuring Residents’ Perceptions of Corporate Social Responsibility at Small- and Medium-Sized Sports Events

**DOI:** 10.3390/ijerph17238798

**Published:** 2020-11-26

**Authors:** Juan Antonio Sánchez-Sáez, Francisco Segado Segado, Ferran Calabuig-Moreno, Ana Mª Gallardo Guerrero

**Affiliations:** 1 Department of Physical Education and Sports, Faculty of Sport, Universidad Católica de Murcia, Av. de los Jerónimos, 135, Guadalupe, 30107 Murcia, Spain; Fsegado@ucam.edu; 2 Department of Physical Education and Sports, Faculty of Physical Activity and Sport Sciences, Universitat de València, C/Gascó Oliag, 3, 46010 Valencia, Spain; ferran.calabuig@uv.es

**Keywords:** corporate social responsibility perceived, well-being, stakeholder theory, residents, sports events, local development

## Abstract

Due to the increase of sports events in local communities, it has become essential to organize such events in a socially responsible way at the environmental, social, and economic levels. The aim of this research was to develop a measurement tool to help determine the degree of social responsibility perceived by residents at small-medium scale sports events, to guide sports managers towards the design of socially responsible sports events. From the elaboration of a questionnaire developed ad-hoc, the perception of the residents was analyzed (*n* = 516). The psychometric properties of the tool, composed of 35 items, were analyzed by means of an exploratory and confirmatory factor analysis. As main conclusions, we were able to contrast the validity and reliability of the questionnaire on the perception of corporate social responsibility in small-scale sports events, around the dimensions of Sustainable Sports Activity, Social Cohesion, and Well-Being. As a consequence, it allowed us to identify three strategic management areas towards which the organizers of these events should focus special attention if they want to progress towards the achievement of socially responsible sports events.

## 1. Introduction

In recent decades, the organization of sports events has increased, with such events integrating themselves into the daily lives of local communities. This has led to various positive and negative impacts on the municipalities that host such events. These impacts can be economic (job creation and price increases), tourist–commercial (improvement of the image of the host locality and inadequate facilities), physical–environmental (the preservation of heritage and ecological damage), social–cultural–sports (civic pride, strengthening of traditions, sports promotion, and crowds), psychological (festive atmosphere and cultural shock), and political–administrative (recognition of other localities or nations and corruption) [1].

Many of the impacts caused by the celebration of a sports event influence the host community of that event [2,3]. This is why the concept of a socially responsible event is fundamental to mitigate or eliminate negative impacts and maximize positive ones [4]. This goal will be achieved not only by being sustainable with regard to the environment but also economically sustainable with respect to the host community. For this reason, the organizing entities should conceive of Corporate Social Responsibility (CSR) in sports as the way in which an entity seeks to align its values and behaviors with those of its stakeholders [2]. Therefore, an approach in which all parties involved in the sports event win: residents, suppliers, organizers, environment, etc.

If the organizers of events wish to achieve the greatest possible success in holding such activities, they must identify each of their target groups as a fundamental premise. In addition, they should consider the needs and expectations of groups and perceive the greatest possible number of them [5,6,7]. The organizing entities should be aware that the setting in which they develop their activity is the community and that a strong network should be created between the organizing entities and the different groups of stakeholders in that space, especially the residents.

Stakeholder Theory [8] is proposed as a way to understand these interest groups in sports events. Different studies focused on sports events, moreover, identified residents as one of the main affected groups [9,10,11,12].

Although the use of Stakeholder Theory and CSR is widely accepted in management literature (e.g., Reference [13,14]), both are still limited in the sports industry. However, Walzel et al. [15] indicated that their use is increasing considerably.

This relationship is providing various lines of research [16], such as analysis of the implementation of CSR in the sports industry. One example is the application of these relationships in professional leagues or sports events [17,18,19,20,21,22,23]. However, most of these works are focused on mega events or large sports events, almost never on determining the Perception of Corporate Social Responsibility (PCSR) of stakeholders in small or medium sport events through a face-to-face survey system [16].

This purpose requires analyzing smaller events that are, in principle, designed to produce minimal negative impacts and high positive impacts, as well as develop the municipality that hosts them through the enhancement of cultural heritage and local customs [24,25,26,27,28,29,30,31,32,33,34,35,36]. In addition, small-medium events are defined as those that competitors can outperform spectators, usually held annually, cause little media interest and generate limited economic activity [27,37]. These characteristics that small- and medium-sized events bring together coincide with the dimensions of CSR proposed by the literature to measure their perception [38,39,40].

To determine the perception of the event as socially responsible by one of the decisive stakeholders, the residents of the host city of the event will be analyzed. In this case, we study the “La Ruta de las Fortalezas” held in Cartagena (Spain), in which characteristics classify the event as Type D or a small–medium size, featuring a regular organization, the same venue, a national level, national competitors, the low presence of spectators, reduced public investment, the use of existing facilities, the presence of local media, limited economic activity, and problems, such as traffic, crowding, or limited access to public areas that are rare or non-existent [37,41,42]. In addition, this sporting event was selected for integrating different socially responsible actions into its organizational program, such as the route of the event that runs through the city’s main heritage sites, the hosting of a university congress, a race adapted for disabled participants, the hosting of environmental waste collection days, and the restoration of historical paths. For this reason, it was appropriate to select this event to determine if the actions implemented by the event are considered by the residents to be socially responsible.

The significance of this study is that it fills in the lack of research on addressing the perceptions of different stakeholders on CSR actions implemented at smaller scale sporting events. The two most relevant references found in the literature are a study by Walker et al. [22] and a study by Walker et al. [43], but these studies focus on mega-events: Beijing 2008 Olympic Games and FIFA World Cup 2010 in South Africa.

This research aims to develop a measurement scale to determine the degree of corporate social responsibility perceived by residents for a sporting event held in their community. This study focused on smaller-scale sporting events for two main reasons: (a) To fill in the scarce literature on the subject and (b) to promote interest in the organization of local events in all cities [44]. Nevertheless, this scale can also be applied to other contexts, such as large events or mega-events.

This paper is composed of 6 sections: Section 1, introduction, in which the main purpose is to contextualize the research. Section 2 is the theoretical framework that establishes the link and relationship between CSR and sports. Section 3 is the materials and methods section, describing the design of the research and the sample selected for the study and concluding with the various measures. Section 4, the results section, shows the relationship between the different variables after their analyses. Section 5, the discussion section, is where the results obtained are compared with the existing literature. The article ends with Section 6, the conclusions section, where the most important findings are highlighted. In addition, implications for management are provided, the limitations found in the investigation process are presented, and future lines of research are determined.

## 2. Theoretical Background

### 2.1. Stakeholders Theory in Sporting Events

Richard Edward Freeman [8] established the theory of stakeholders as a strategic management theory to aid the sustainability of organizations.

Several reasons make this theory suitable for our study. This theory postulates that an organization must be understood in a pluralistic way. Therefore, an organization is not only comprised of its shareholders and/or workers but must also be understood by the agents it affects and those who affect the organization. Furthermore, with this business perspective, the relationships established between the different stakeholders are no longer only economical but also introduce a moral relationship that yields the expectation of ethical behavior among the stakeholders. These factors can lead a company to commit to social responsibility, which is very pertinent for organizations that run small-scale sports events.

This theory defines stakeholders as any group or individual that can affect or be affected by the achievement of a company’s objectives [45]. From this point of view, it is clear that the support of residents towards the celebration of sports events in their community affects the viability and impact of such events. Therefore, maintaining a good relationship with stakeholders is beneficial for both parties [46].

When the intention of a stakeholder is to continue to support the company, it is more likely that the company will achieve its performance objectives and give positive feedback for that relationship. On the other hand, when a stakeholder who is dissatisfied with the company experiences a condition of disruption and no longer perceives a psychological link to the company, the stakeholder may behave in a destructive or counterproductive way to the company [47]. An example of the latter situation can be found in the Rio de Janeiro Olympics, where there was a significant public response that led to a significant loss of prestige and a decrease in all types of possible benefits [48].

The use of stakeholder theory is, therefore, very relevant to this research and fully justified given the close relationship between these stakeholders (i.e., residents and organizers). Working together and uniting the different stakeholders has proven to be very beneficial for the community [49]. This mutual benefit is greater if there is full collaboration with local stakeholders. In the present case, collaboration is especially important given the local character of the event, which means that the negative effects of overcrowding are not perceived and can even be accepted [50].

### 2.2. CSR in Sporting Events

CSR is increasingly being implemented in sports organizations [51], but, in the private sphere, CSR is acquiring a business dimension with strong links that support not only economic interests but also the social and environmental commitment demands required by the community [6,16,52]. 

Given the reality of sports, and in analogy with The Copenhagen Charter (1988), some authors have highlighted the Olympic Charter itself (1978) as one of the first documents to bring together socially responsible characteristics in the field of sports [53]. Indeed, the Olympic movement has its roots in social commitment.

Many sports entities have confused the term CSR with event sponsorship, patronage, non-refundable donations, etc., associating such actions solely with obtaining economic benefits in terms of competitive advantages [21]; however, the stakeholders themselves (supporters, residents, etc.) expect other achievements from sports entities, such as socially responsible commitments.

The role that CSR plays in sports is different from that of other industries due to the transversal nature of sport in relation to aspects, such as integration, equality, environmental protection, the promotion of culture, and the enhancement of heritage [21,46]. All entities, including sports entities, have the responsibility to understand, through an evaluation of their impacts, how they affect society in general and residents in particular [54]. 

Sports events are conceived of as a business activity that takes place in a specific context—the community. In this way, a strong relationship is created between the organization sponsoring the event and the local communities. This relationship is fundamental to the success of the event, ensuring that sponsors reaching beneficial agreements with local and state governments and try to integrate the sports event within the culture of the local community, thus providing a sense of belonging to the event [34,46,55,56,57]. Sports events, therefore, present a unique opportunity for the development and promotion of CSR initiatives carried out by their organizing committees, thanks to their resources and media exposure.

It is, therefore, essential to consider all of the different proposals for the dimensionality of CSR when planning a sports event. The relationship between the impacts caused by a sports event, both positive and negative [58], and the different dimensions of CSR proposed in the literature to measure their perception is evident [38,39,40]. Therefore, it is necessary to apply the following actions to the programs of sports events (as perceived by stakeholders): the creation of employment, local development (economic dimension), waste management, and energy efficiency (environmental dimension); concern for people by considering their special needs; coexistence with local residents, suppliers, and spectators; and ensuring the protection of human rights (social dimension) [53].

Therefore, interest groups need to know the PCSR (in this study, the PCSR of the residents), so that the sports event be perceived as socially responsible and reach the highest possible success [55,59].

From a marketing perspective, CSR has been considered an effective tool to improve the image of companies [60]. Furthermore, meeting social objectives, building customer loyalty, and developing a responsible brand under the perception of the host community can help soften some of the criticisms around a sports event [61,62]. However, it should be borne in mind that, depending on the countries of origin and cultures of the various stakeholders, there are differences in the perception of CSR. Therefore, for each event, the stakeholders in question and the indicators of the sports product to be analyzed must be identified [22]—in this case, small-medium-scale sports events.

Sporting activities are considered a natural partner for corporations in the presentation of CSR initiatives implemented in this sector. To explain this phenomenon, Levermore [63] offers three main arguments: (a) Sport is an entity that connects with many grassroots communities where a business might have had difficulty becoming established; (b) sport allows the creation of a common ground where society and organizations can work together [59]; and (c) sport programs provide a natural setting where collaborating partners and civil society can meet and strengthen their business operations [20].

These reasons are why the integration of CSR in companies through sports is a powerful means by which to offer a return on actions that benefit the community. Indeed, sports organizations are already implicitly woven into society, an integrative feature that commercial business organizations do not possess [64]. Because of this, the binomial formed by CSR and sport could be used to meet the needs of residents. In this way, sport could be offered as a bridge between the social and economic gaps in the community [63].

To resolve this situation, sports events can implement actions within their organizational objectives that highlight the historical and cultural heritage of a population, including people at risk of social exclusion in the organization [53] or those that promote the protection of the natural environment within the rules of the event itself. Sports events can thus create a network of relationships between sports organizations, governments, and local associations that are fundamental to the understanding of residents’ needs [6].

Thus, the implementation of this type of initiative in sports bodies, whatever their nature, yields a positive increase in their reputation and image for not only local and national but also international communities, thereby increasing their value as a brand and giving their intangible resources value alongside their financial and tangible resources [30,61,62,65,66]. CSR in sports can play a very important role in influencing trends in stakeholder attitudes and behaviors, thereby bringing them closer together.

Therefore, the transversality of sports causes CSR to be included in each action or process for the celebration of sports events. Consequently, CSR in sports events can be defined as actions adopted in a voluntary way by the organizing committee that use the transversality of sports to try to satisfy the expectations of the greatest possible number of stakeholders, especially the local population of the area where the event is held, thereby achieving common objectives by mitigating negative impacts and trying to boost the positive ones in both the present and the future.

Consequently, we propose the following research hypothesis: “Stakeholders’ perception of cooperative social responsibility in small–medium-sized sports events is multidimensional and around Sustainable Sports Activity, Social Cohesion, and Well-Being”.

## 3. Materials and Methods

### 3.1. Design and Sample

**Figure 1:**
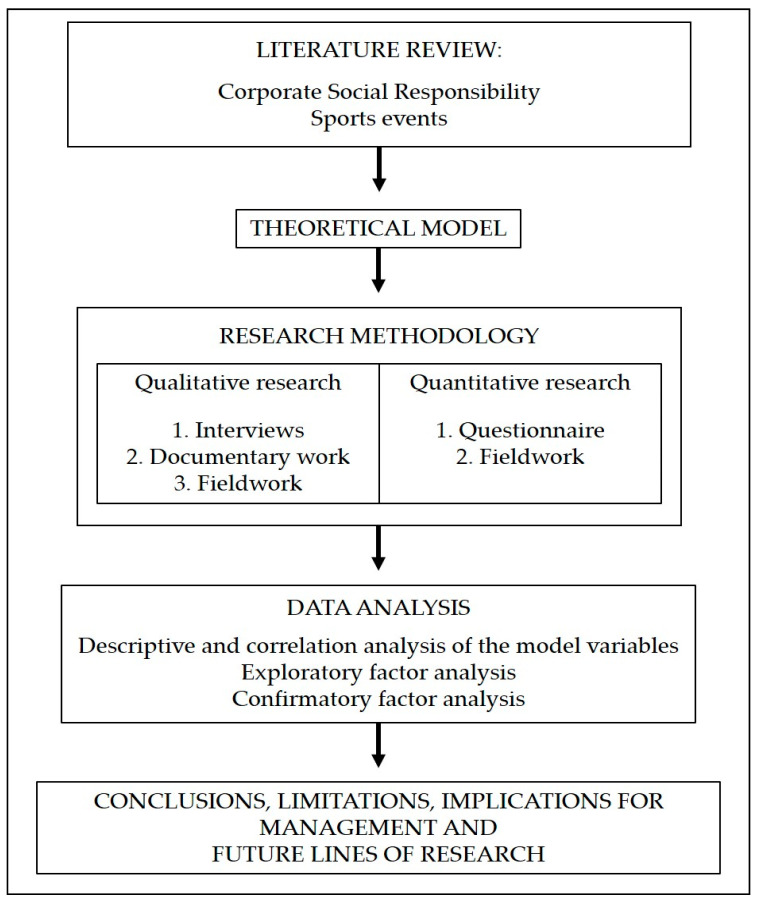
Phases of research development.

The methodology applied to this research followed the recommendations of different authors in each of its phases [67,68,69,70]. We began by establishing a theoretical framework to determine the state of the topic to be studied: Corporate Social Responsibility and the celebration of sports events. During the research process, qualitative and quantitative techniques were used at different stages. A documentary analysis of “La Ruta de las Fortalezas” was also carried out to gather information about the event (the history of the event, the organizers, the itinerary, parallel activities, etc.), with the aim of contextualizing the research (Figure 1).

**Figure 2:**
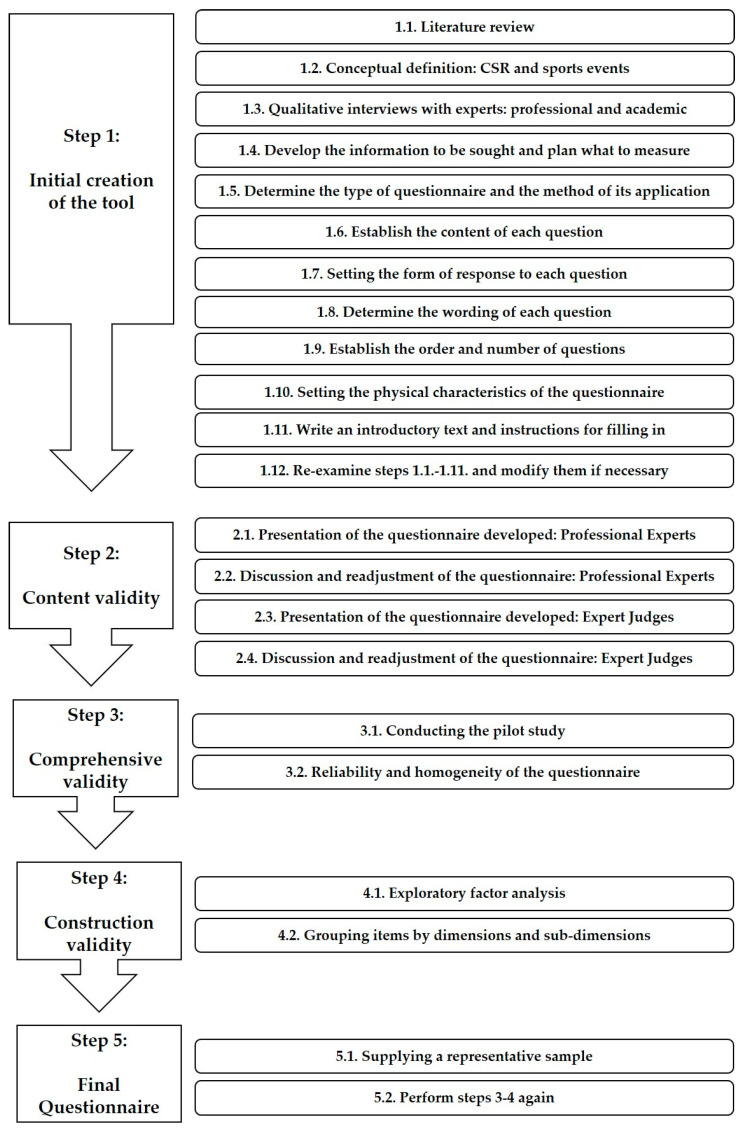
Procedure for the elaboration and validation of the questionnaire.

The criteria for the development and validation of the questionnaire were based on five steps marked by specific publications [30,69,71,72,73] (Figure 2).

For the development of this tool, we used Structural Equations Modeling (SEM). SEM allowed us, on the one hand, to evaluate the measurement quality of the set of variables used to measure the PCSR’s latent construct and, on the other hand, to evaluate the relationships between variables [74]. For this purpose, we used the ESQS software 6.3 [75] for the Confirmatory Factor Analysis (CFA) and SPSS 21 for the Exploratory Factor Analysis (EFA).

Before arriving at the final questionnaire, we followed two phases: qualitative and quantitative. (A) Qualitative analysis of the pilot questionnaire; for the qualitative design, the initial version of the questionnaire was submitted for evaluation by a committee of experts made up of professionals in sports events management, the practical application of the CSR, and university professors and researchers in CSR [70,73,76]. The criteria for inclusion of these committees were more than 5 years of experience in their field and active participation in areas related to sports management and/or CSR. The committee was made up of a total of 18 members. The average age of this group of specialists was 41.2 years, and they had an average of 14.6 years of experience in the sector (professionals and university professors). The participants were requested to issue an assessment of the items, the dimensions, and the sub-dimensions, as well as the questionnaire, in a global way over four phases; the results were collected in a registration sheet. This assessment was first made from a qualitative perspective, where the participants were asked to make all the comments and contributions that they considered appropriate. (B) Quantitative analysis of the pilot questionnaire; an assessment was made using a quantitative analysis. In this case, all the items were evaluated by the committee of experts by analyzing their relevance, clarity, specificity, and significance, as well as the relevance of the item to the dimension and sub-dimension [77]. All questions used a Likert-type scale (1 “strongly disagree”/7 “strongly agree”), where expert judges gave their degree of agreement on whether or not to maintain the item [78]. All items complied with the considerations of Cristobal et al. [79], with no items being eliminated since all items obtained an average above 4 (the minimum was 5).

After delivering the questionnaire composed of 44 items related to the PCSR, the version for the pilot study was determined. Once the results of the pilot study were obtained, the names of the factors and the sub-dimensions were changed to reflect the subject matter of the attributes that make up each of them. In addition, the items that saturated the same factor were regrouped, and the items that did not reach the minimum established value of factor saturation were eliminated (0.40). The final result for the PCSR implemented in “La Ruta de las Fortalezas” included 35 items in three dimensions.

Of the total population (58,483 inhabitants), 524 personal surveys (face-to-face) were carried out using an intentional-non-probability sampling procedure segmented by gender with a 95% confidence interval. The choice of this sampling procedure over others was motivated by the absence of a census of permanent residents in the city of Cartagena, as this number is different from the number of registered inhabitants, which is commonly used in this type of research (e.g., Reference [4,80,81,82,83]). However, although this type of sampling is not representative of the general population, an attempt was made to collect the existing ratio between men and women, as well as the different age ranges from 18 years onwards. In addition, the sample was stratified by gender and age quotas. However, to make the sample as representative as possible and reduce the bias inherent in this type of sampling, Sudman’s [84] recommendations were followed when selecting the population under study. In this way, the following conditions were considered: (a) Surveys were conducted in four neighborhoods in the urban area of the city of Cartagena; (b) within each of the neighborhoods, different times of day and dates were alternated to ensure variety in the sampling period at the same location; and (c) different quotas of residents were used based on the ages and genders of the inhabitants.

A total of 8 surveys were rejected due to errors or omissions in the responses, and 516 were ultimately considered valid. The gender distribution was 260 for women (50.40%) and 256 for men (49.60%). The majority segment was between 35 and 49 years of age (30.04%). The vast majority had lived 9 years or more in the locality where the event under study was taking place (92.83%). This aspect, together with the fact that 100% of the sample claimed to know or have heard of “La Ruta de las Fortalezas” ensured a response with a minimum degree of knowledge about the selected sports event. In addition, the level of education in the sample was as follows: 32.75% had a bachelor’s degree/higher vocational training or equivalent; women respected this trend (33.46%) but not men, among whom 32.42% had university studies. In both the total sample and the sub-samples, the bachelor’s degree/higher vocational training or equivalent and university options represented more than 59% of those surveyed. In addition, the employment situation of those surveyed showed that 26.74% were employed by private companies. Women responded in the same way to employment status with 31.92%, while 25.78% of men were retired or pensioners. Finally, it should be noted that 92.83% of those surveyed lived in the city for more than 9 years. This aspect ensured that residents were aware of both the event and the local culture.

### 3.2. Measures

All the scales used in the questionnaire were taken from the literature [4,22,55,85,86,87,88,89,90,91] and adapted to the context of the current research (Table A1 in Appendix A). The scale used to measure the perception of the residents of Cartagena regarding the CSR actions implemented in “La Ruta de las Fortalezas” was composed of 35 items seeking to measure the population’s perception of the CSR of the selected event. These items are grouped around 3 dimensions and 6 sub-dimensions. The 3 dimensions around which we have structured the PCSR are “Sustainable Sports Activity”, “Social Cohesion”, and “Well-being”.

The “Sustainable Sports Activity” dimension contains 15 indicators distributed in the following sub-dimensions: sports promotion (6 items), conservation (3 items), and quality of life (6 items). The attributes represented are sports for all (which includes different types of populations), the encouragement of physical activity, the promotion of good environmental sports practices, cultural exchange, and tourism development [1,26,27,35,54,72,75,76]. 

The “Social Cohesion” dimension includes 13 indicators distributed in the following sub-dimensions: heritage (6 items), economic (3 items), and education/training (4 items). The attributes represented are the enhancement and conservation of historical and cultural heritage, the promotion and development of local trade, investment in sports, and the promotion of cultural activities [1,21,22,64,88,92,93]. 

The “Well-being” dimension contains 7 indicators that make up a single sub-dimension representing aspects that disturb the population’s daily routine, entertainment, and environmental conservation [1,21,64,88,92]. 

All responses were measured on a 7-point Likert scale, where 1 indicates total disagreement and 7 indicates total agreement with the statement.

Before the estimation of the structural model, it was necessary to assess the psychometric properties of the scales. To achieve this goal, an EFA (construct validity) was performed using the Varimax principal component method (≥0.40). To analyses the reliability of the proposed scale, the Cronbach’s α value was calculated [1]. After obtaining these data, the first-order CFA and a subsequent second-order CFA were carried out to ascertain whether a higher concept or PCSR exists behind the three dimensions. One of the prerequisites for the analysis of a structural model is confirmation that the latent variables or constructs are adequately measured, so it is necessary to test the measurement models [93]. Thus, in this study, the measurement model for the perception of the local residents regarding the implementation of CSR actions in sports events was tested. The following adjustment indices were used to assess the suitability of this model: the Bentler–Bonnet Non-Normed Fit Index (NNFI), the Comparative Fit Index (CFI), the Incremental Fit Index (IFI), the Standardized Root Mean Square Residual (SRMR), and Root Mean Square Error Approximation (RMSEA).

## 4. Results

### Measurement Quality and Relationship between Variables

The Kaiser-Meyer-Olkin index sample adequacy test (KMO = 0.898) and the Barlett sphericity test (χ^2^ = 7593.306; gl. = 595; *p* < 0.00) indicated good construct validity. Once the rotation of the factors was performed, the items were grouped into three dimensions. All items obtained loads greater than 0.40, except for one attribute, “well-being4” (0.388), which was maintained at the expense of further analysis. Most variables saturated a single factor that corresponds to the proposed dimensionality. The distribution of the items was logical. Given the results of the data obtained, factorial validity was able to be interpreted.

The resulting correlations (Cronbach’s α) were higher than those recommended by different authors [1], reaching a value of 0.903. For each one of the established dimensions, the “Sustainable Sports Activity” factor reached a Cronbach’s α of 0.870, the “Social Cohesion” factor reached 0.874, and the “Well-being” factor reached 0.783. In this way, the stability and high internal consistency of the scale was ensured.

**Figure 3:**
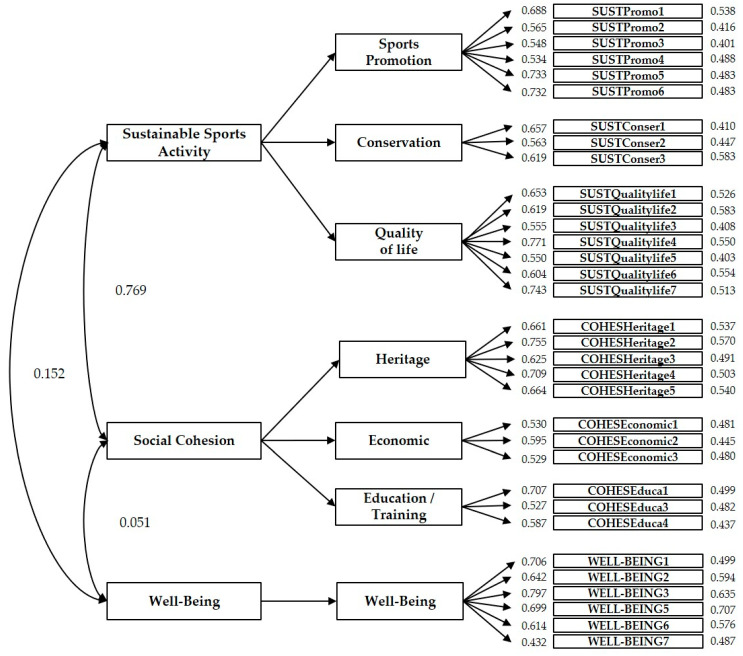
First order (2nd phase) Confirmatory Factor Analysis (CFA) model with the weights of each factor and the covariances between factors of the explained variance.

An adequate model fit is indicated by values greater than 0.90 on the NNFI and CFI indices and less than 0.05 on the SRMR and RMSEA indices. However, different authors using the RMSEA index indicate that values lower than 0.08 are acceptable [3,22,83,88,90,94,95,96]. All the presented indices (except the SRMR index— Lagrange Multiplier test) were obtained through a Robust method (Yuan–Bentler Correction). This procedure is used when multivariate kurtosis values suggest that the sample does not have a normal distribution, as in our case (Mardia’s normalized coefficient = 77.28) [74]. Figure 3 shows the final model obtained after several adjustments. This model was ultimately composed of three dimensions and 33 items after the two-stage setups. In this final model (2nd phase), the items COHESEduca2 of the “Social Cohesion” dimension and the item WELL-BEING4 of the “Well-being” dimension were eliminated. Likewise, the location of the item COHESHeritage6 was modified and relocated to the “Quality of Life” sub-dimension belonging to “Sustainable Sports Activity”. The CFA indices after these modifications show that the model fits the data correctly (phase 1, with the 35 items and without relocation of the item “COHESHeritage6”/phase 2, the final model of 33 items with relocation of the item “COHESHeritage6) [73]: Phase 1: S-Bχ^2^ (524) = 2436.13, *p* < 0.001; * CFI = 0.72; * IFI = 0.73, * RMSEA (90% CI) = 0.071 (0.067–0.064); SRMR = 0.074. Phase 2: S-Bχ^2^ (474) = 882.02, *p* < 0.001; * CFI = 0.91; * IFI = 0.91, * RMSEA (90% CI) = 0.041 (0.037–0.045); SRMR = 0.058. The results confirm the existence of the three factors initially proposed: “Sustainable Sports Activity”, “Social Cohesion”, and “Well-being”. Figure 3 shows the final model of the first order CFA with the factor loads of each attribute, the covariances between factors, and the explained variance.

**Figure 4:**
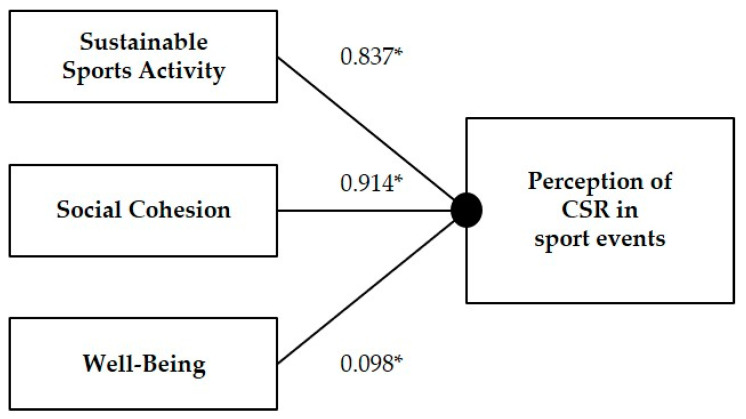
Proposed structural model for the perceived CSR in sports events. * *p* < 0.05. CSR: Corporate Social Responsibility.

The second-order factor analysis confirmed the structural model proposed for the perception of CSR in sports events (Figure 4). As can be seen, the coefficient β resulting from the standardized solution demonstrates that all factors have a positive and significant impact on the perceived CSR at sports events. The “Social Cohesion” factor has the greatest impact (β = 0.914) on the perception of CSR. The second factor by impact importance is “Sustainable Sports Activity” (β = 0.837). The factor with the least impact on the perception of CSR in sports events is “Well-being” (β = 0.098).

## 5. Discussion

The literature on the perception of different attributes of CSR [95,97,98,99] and applying CSR in the sports sector [22] established that CSR has different dimensions. Specifically, there are various investigations with different orientations that theoretically [38,100] or empirically [53] demonstrate the multidimensionality of CSR [98].

The difficulty encountered by this study when creating a scale for measuring the perception of CSR at smaller sports events was trying to establish the connections of attributes from studies attempting to determine the perception of residents at sports events [35,83,92,101,102] and studies on the perception of CSR in sports. Introducing CSR in the field of sports meant incorporating adapted indicators that reflect the link between both aspects, such as the indicators used by Sheth and Babiak [21].

The construct validity of the proposed dimensions, “Sustainable Sports Activity”, “Social Cohesion”, and “Well-being”, and of the scale in general, were corroborated by the values of the sample adequacy, Bartlett’s sphericity, and multivariate normality [103]. Thus, the methodology used in other works for determining the perception of residents based on some attributes was successfully followed to analyze the validity of the measurement scales through the EFA and the CFA [3,66,88,92,93,96,103,104]. This analysis of the data was used to verify the appropriateness of the measurement scale, thereby surpassing other research that only used the EFA [55,82,101,105].

The first-order CFA highlighted the need to eliminate two items. In relation to the second order CFA, this confirmed the multidimensionality and existence of a latent variable: CSR perception in sports events. These attributes are better than others presented in other studies that asked questions that were impossible for residents to answer, since they focused on aspects related to finances, environmental protection protocols, workers’ rights, offering continuing education to employees, reducing the amount of waste, or being economically viable [21].

The items finally included in the questionnaire for each of the dimensions represented the following attributes, in line with different investigations: “Sustainable Sports Activity”—sport for all, encouragement of physical activity, promotion of good environmental sports practices, cultural exchange and tourism development [55,85,86]; “Social Cohesion”—conservation of the historical and cultural heritage of the town, development of local trade and public investment in sport and cultural activities [4,22,106,107]; “Well-being”—negative impacts and entertainment and environmental conservation [55,82,89].

In relation to the objective of this research, similar studies have shown the important practical implications of knowing the degree of perception of corporate social responsibility implemented by entities in their activity [22,85,86,107]. In this respect, two types of implications could be identified: (A) Strategic, such as improving the reputation of organizations [107] or increase in brand value [108,109]; (B) Awareness raising, to determine strategies to meet the expectations and needs of the residents [110] or to promote the local development of the host community [111]. In contrast, Misener [112] explains that the application of CSR actions sometimes conflicts with the achievement of the entities’ primary objectives for the development of events. These actions are used as part of the social marketing of the event to positively influence the perception of different stakeholders and reduce criticism of the event. Therefore, for sports event managers, this information is very valuable in order to improve the positive results of the event.

Finally, it should be noted that, in the context of sporting events, the study of the relationship with CSR has focused on improving the image of the organizing entity and on the perception of consumers, sports tourists or sports managers of mega-events, such as: Beijing 2008 Olympic Games [22], FIFA World Cup 2010 in South Africa [43], or London 2012 Olympic Games [113]. In this sense, this research focuses on the smaller events, not on their organizing entity, and on the perception of local community residents, as they are considered to be one of the key stakeholders [9,10,11,12].

## 6. Conclusions

At the beginning of the research, the following hypothesis was proposed, “Stakeholders’ perception of cooperative social responsibility in small–medium-sized sports events is multidimensional and around Sustainable Sports Activity, Social Cohesion, and Well-Being”, and, after its confirmation, we can conclude that:

- The present research contributes methodologically by offering a valid and reliable multidimensional scale of measurement for the perception of CSR in sports events from the perspectives of residents on 33 items composed of three dimensions: “Sustainable Sports Activity” (16 items), “Social Cohesion” (11 items), and “Well-being” (6 items).
- These dimensions resulting from the CFA combine the indicators necessary to determine the residents’ perceptions of CSR implemented in a sports event. Actions linked to “Sustainable Sports Activity”, such as sports for all, the encouragement of physical activity, the promotion of good sports practices in the natural environment, cultural exchange, tourist development, etc.; “Social Cohesion”, such as promoting the value and conservation of historical and cultural heritage, the encouragement and development of local trade, investments in sports, the promotion of cultural activities, etc.; and “Well-being”, such as minimizing negative impacts, such as pollution, traffic congestion or roadblocks, entertainment, environmental conservation, etc., could, if integrated into the planning and organization of the event, make the event socially responsible.
- The final objective of this scale is to determine the perception of local residents on the CSR actions implemented in a small–medium scale sports event. It is important to highlight the difficulty of not having reference studies, so these results are the foundation for continuing this line of research.

### 6.1. Managerial Implications

Primarily, management provides a valid and reliable tool for measuring residents’ perceptions of CSR actions at sports events. In this way, it becomes possible to determine the effectiveness of the socially responsible practices implemented by the organization. As a result of analyzing these data, useful information can be obtained for decision-making by the organizers. In addition, strategies can be established to incorporate the various stakeholders (once identified) represented by collectives into the organization’s committees. It will also be possible to collect the different sensitivities of these groups and thus involve them in decision-making (even if they are simply advisory bodies), thus responding to their needs. In this way, the involvement of these groups will be ensured, as well as support for the event. The ultimate aim will be to seek socially responsible local development through the celebration of sports events as elements of territorial structuring, thereby promoting the city’s own culture as an inherent part of the event (monuments, gastronomy, and traditions) or even recovering disused spaces and uninhabited rural areas as the setting for the event. All these strategies, aimed at creating a socially responsible environment, contribute to increasing the influence of the organizing entities (companies, clubs, federations, etc.) in society by transmitting social, environmental, and local development values.

### 6.2. Limitations and Future Research Lines

While the actions that make up the concept of CSR are widely perceived by society and studied by researchers, the CSR construct and its perception is more recent and therefore has limitations when researching in very specific contexts, such as small sporting events [15]. However, the last decade has seen an increase in interest among researchers in this field of study, which explores the role of sports in promoting social and community development [16,21,63,105]. However, within this sport–CSR binomial, a small part of this research is oriented towards the use of CSR in conjunction with sports events for social development [16,63,105].

As a line of future research, we propose to extrapolate this research, framed in the smaller sporting events, in contexts of similar events of national or international character, including all its interested groups using the model of identification of the stakeholders proposed by Xue and Mason [7], as a starting point. It is also considered interesting to compare the perceptions of different cultures, as Maignan [114] indicates, about the concept of CSR applied in small- or medium-sized sports events. It would also be very interesting to produce a short version of the scale. In this way, the scale would be more likely to be compared against other constructs and applied with less effort at sporting events.

A larger study that considers other stakeholders (e.g., politicians, local entrepreneurs, and organized civil entities) and relates the perceived impacts on the reputation of the organizers may be another fruitful line of research for small events. If these stakeholders were also involved in decision-making related to the organization of the event, as suggested by Bazzanella et al. [115], an experimental study could be developed to determine if such involvement is conducive to greater support for the event and the image of the company.

## Appendix A

**Table A1:** Scale of perception for corporate social responsibility in small–medium-sized sports events.

Items	
1	“La Ruta de las Fortalezas” promotes sports among youth.
2	It promotes the participation of women in sports.
3	It promotes sports participation in adulthood (30–40 years of age).
4	It promotes and increases knowledge about local sports clubs.
5	Interest in mountain running/athletics/physical activity is increasing among residents.
6	It increases my knowledge about the organization of this type of race.
7	It helps one positively consider the environmental protection of the territory.
8	“La Ruta de las Fortalezas” respects the conservation of natural resources.
9	It serves as a stimulus for the recovery and maintenance of historic roads and trails.
10	It contributes to improving the hospitality and solidarity of the city with the assistants and participants of the event.
11	I feel like I own the event.
12	It provides an opportunity to meet new people.
13	Celebrating this event in my city helps me feel good about myself and society as a whole.
14	This event improves my quality of life.
15	It attracts tourists to Cartagena.
16	“La Ruta de las Fortalezas” increases my pride as an inhabitant of Cartagena.
17	Through the event, the cultural heritage of the town is valued.
18	“La Ruta de las Fortalezas” serves as a stimulus for the preservation of local culture.
19	It provides an incentive for the restoration and maintenance of historic buildings.
20	It increases the knowledge of the cultural and historical heritage of the town.
21	The celebration of “La Ruta de las Fortalezas” encourages the promotion of local traditions.
22	It provides an opportunity for local business.
23	It helps generate opportunities for employment.
24	The celebration of “La Ruta de las Fortalezas” contributes to increasing the investments of the City Council in sports.
25	The educational activities (Congresses, etc.) of “La Ruta de las Fortalezas” culturally enrich the population of Cartagena.
26	It promotes the inclusion of groups under risk of social exclusion.
27	It promotes volunteering for the celebration of other events in Cartagena.
28	It makes it difficult for residents to access public spaces, streets, or roads.
29	It causes an increase in the prices of some products, such as food and/or services, in the locality.
30	It causes alterations in the population’s daily life (e.g., noises, traffic jams).
31	It causes the agglomeration and/or disorderly accumulation of people in the city.
32	This event causes noise pollution.
33	During its course, the event causes the deterioration of the environment (e.g., pollution, rubbish, erosion, etc.).
